# Implementing accountability for reasonableness framework at district level in Tanzania: a realist evaluation

**DOI:** 10.1186/1748-5908-6-11

**Published:** 2011-02-10

**Authors:** Stephen Maluka, Peter Kamuzora, Miguel SanSebastián, Jens Byskov, Benedict Ndawi, Øystein E Olsen, Anna-Karin Hurtig

**Affiliations:** 1 Institute of Development Studies, University of Dar Es Salaam, P.O. Box 35169 Dar Es Salaam, Tanzania; 2 Umeå International School of Public Health (UISPH), Umeå University, SE 90185 Umeå, Sweden; 3 DBL-Centre for Health Research and Development, Faculty of Life Sciences, University of Copenhagen, Thorvaldsensvej 57, DK 1871 Frederiksberg, Denmark; 4 Primary Health Care Institute (PHCI), P.O.Box 235, Iringa, Tanzania; 5 Haydom Lutheran Hospital, Mbulu, Manyara, Tanzania

## Abstract

**Background:**

Despite the growing importance of the Accountability for Reasonableness (A4R) framework in priority setting worldwide, there is still an inadequate understanding of the processes and mechanisms underlying its influence on legitimacy and fairness, as conceived and reflected in service management processes and outcomes. As a result, the ability to draw scientifically sound lessons for the application of the framework to services and interventions is limited. This paper evaluates the experiences of implementing the A4R approach in Mbarali District, Tanzania, in order to find out how the innovation was shaped, enabled, and constrained by the interaction between contexts, mechanisms and outcomes.

**Methods:**

This study draws on the principles of realist evaluation -- a largely qualitative approach, chiefly concerned with testing and refining programme theories by exploring the complex interactions of contexts, mechanisms, and outcomes. Mixed methods were used in data collection, including individual interviews, non-participant observation, and document reviews. A thematic framework approach was adopted for the data analysis.

**Results:**

The study found that while the A4R approach to priority setting was helpful in strengthening transparency, accountability, stakeholder engagement, and fairness, the efforts at integrating it into the current district health system were challenging. Participatory structures under the decentralisation framework, central government's call for partnership in district-level planning and priority setting, perceived needs of stakeholders, as well as active engagement between researchers and decision makers all facilitated the adoption and implementation of the innovation. In contrast, however, limited local autonomy, low level of public awareness, unreliable and untimely funding, inadequate accountability mechanisms, and limited local resources were the major contextual factors that hampered the full implementation.

**Conclusion:**

This study documents an important first step in the effort to introduce the ethical framework A4R into district planning processes. This study supports the idea that a greater involvement and accountability among local actors through the A4R process may increase the legitimacy and fairness of priority-setting decisions. Support from researchers in providing a broader and more detailed analysis of health system elements, and the socio-cultural context, could lead to better prediction of the effects of the innovation and pinpoint stakeholders' concerns, thereby illuminating areas that require special attention to promote sustainability.

## Background

Attempts to strengthen district-level planning and priority setting in Tanzania are mainly based on burden of disease measures, cost-effectiveness, and related planning tools, and have not achieved adequate and sustainable improvements [1,2]. National health policies and guidelines promote more inclusive planning processes, but concrete involvement of stakeholders in the actual planning and priority-setting process is still limited [3-6]. Innovative approaches to priority setting that fairly reflect, not only the mainly provider-defined burden of disease, but also incorporate capacities and interests of all stakeholders are required. In the light of this, researchers from the Primary Health Care Institute, the Institute of Development Studies in the University of Dar es Salaam, and the National Institute for Medical Research in Tanzania, in collaboration with other research institutions from Europe, launched a five-year project called, Response to Accountable Priority-Setting for Trust in Health Systems (REACT). The objectives of the REACT project are to strengthen the legitimacy and fairness of priority setting through implementing the Accountability for Reasonableness framework (A4R) in Mbarali District in Tanzania, Malindi District in Kenya and Kapiri Mposhi District in Zambia, and to evaluate subsequent changes in the quality, equity and trust of health services and interventions [7].

The A4R framework consists of four conditions: relevance, publicity, appeals/revision, and enforcement [8-11]. Relevance requires that decision makers should provide a reasonable explanation of how they seek to meet the varied healthcare needs of a defined population within available resources. Specifically, a rationale will be 'reasonable' if it sets out evidence, reasons, and principles that are generally accepted as relevant by society. Publicity is the requirement that decisions are made by a group of decision makers, and that the rationales for priority-setting decisions be made accessible to the wider public and open to scrutiny. The appeals/revision condition is an institutional mechanism that provides stakeholders with an opportunity to challenge and revise decisions in the light of new evidence. Finally, enforcement entails organisational leadership and public or voluntary regulation of the decision-making process to ensure that the first three conditions are met.

However, while the A4R framework acts as a guide to achieving a fair and legitimate priority-setting process [12-15], our understanding of the processes and mechanisms that determine its degree of success in the achievement of fairness and legitimacy (and its impact on quality, equity, and trust) remains largely an open question [16]. Priority setting takes place within the complex system of healthcare delivery, which consists of layers of social actors, social processes, and structures: in its decision-making processes, the district health decision makers deal with many different actors; multiple agendas need to be reconciled in the planning and priority-setting process in the district; priority-setting decisions are determined by guidelines from the central government; decisions are influenced by the cultural norms and values of the involved actors -- these include not only those values medically- and otherwise technically-defined (such as burden of disease or cost-effectiveness) but also the local values of the people and institutions involved in setting priorities [17]; and, finally, the decision-making process is influenced by power relations and interests. Power differences in priority setting may be characterised by a mixture of individual wealth, professional status, access to knowledge, authority, or gender, but they are strongly related to the organisation and structure within which the individual actor works and lives [18].

Interventions that seek to influence change in this type of context are generally complex and dynamic; often evolving in response to local circumstances, target-group engagement and other events beyond the control of the implementers, which can adversely affect the impact of the intervention [19]. This paper presents the experience of implementing the A4R framework in Mbarali District, Tanzania in order to find out how the innovation was shaped, enabled and constrained by the interaction between the contexts, mechanisms and outcomes.

## Methods

### The Study Setting

The study was conducted in Mbarali district, in the Mbeya region of Tanzania. Mbarali district was selected by the REACT project, as it is a typical rural district in Tanzania. In Mbarali, like in other districts in Tanzania, the health system is administered by two different central government departments: The Ministry of Health and Social Welfare (MoHSW), and the Prime Minister's Office Regional Administration and Local Government (PMORALG). The MoHSW is responsible for developing policies, monitoring disease patterns, the quality of health services, providing technical support as well as mobilising and supplying resources. The PMORALG deals with the implementation of health policies and monitors the use of funds.

At the district level, the mandate to develop health plans and budgets has been placed under the Council Health Management Team (CHMT). CHMT members are required to work closely with user committees and boards to develop plans and budgets to incorporate them into the Comprehensive Council Health Plan (CCHP) on a yearly basis. The CHMT prepares the CCHP, which is then submitted to the Council Health Service Board (CHSB). The CCHP identifies areas of priority, based on locally available epidemiologic data and health service statistics, in light of a nationally defined essential health package (EHP) and charts out activities to be undertaken on an annual basis (Table 1).

**Table 1:** Priority areas contained in the district health plans

	Priority Area	Disease control and activities to be implemented
1	Reproductive and Child Health	Antenatal care, obstetric care, postnatal care, family planning, integrated management of childhood illness, immunisation, post-abortion care, nutritional deficiencies.

2	Communicable disease control	Malaria, TB/leprosy, HIV/AIDS, epidemics (cholera, meningitis, yellow fever, measles, polio).

3	Non-communicable disease control	Acute and chronic respiratory, cardiovascular disease, neoplasm/cancer, injuries/trauma, mental health, drug abuse, anaemia and nutritional deficiencies.

4	Treatment of other common diseases of priority within the district	Eye disease, oral conditions, skin disease, schistosomiasis, plague, relapsing fever.

5	Community health promotion	Health communication for behaviour change; water, hygiene and sanitation; school health promotion; food control and hygiene; occupational health & safety; enforcement of by-laws and regulations related to health.

6	Strengthen organisational structures and institutional capacities at all levels	Council health service board and health facility governing committee functions, utilities management, health management information systems, capacity development for human resources, public and private collaboration, and supportive supervision and inspection.

Once the CCHP is approved by the CHSB, it is submitted to the Full Council, which is the highest political body in the district. Having been approved by the Full Council, the CCHP is submitted to Regional Secretariat, which assesses plans and reports with respect to compliance with national guidelines. The completed district health plan is submitted to the PMORALG and MoHSW for final approval. After the budget review process in Parliament, funds are then disbursed, often drastically modified [20-22]. Moreover, once the money has been allocated, the districts cannot change what it is to be spent on without higher-level approval, otherwise it is regarded as a violation of financial regulations. In addition to the funds from the central government, the CHMT may get funds from other sources, such as community health funds (obtained from cost-sharing) and from district councils (which are often unreliable) and from other agencies and donors.

### The REACT project in Tanzania

REACT is a five-year European Union funded project, aimed at testing the application and effects of the A4R framework in Mbarali District, Tanzania. The REACT research process involves the application of A4R, a scientific assessment of this process, as well as an evaluation of the applicability of its conditions to priority setting and its subsequent effects on health systems [7]. A preliminary phase of the implementation of the A4R framework in the district began in 2006, involving gathering baseline data, consultation and planning. The full application of A4R began in 2008, and the project will end in December 2010.

The research-based improvement in Mbarali district combines three linked methods: case study research to describe priority setting, interdisciplinary research to evaluate the description against A4R, and action research to improve priority setting in context [23].

To meet its goals, the REACT intervention employs three overlapping strategies: active collaboration with district health decision makers, sensitisation workshops with stakeholders, and presence of a project focal person in the district to facilitate and document the implementation process.

First, the process of change in the district is carried out by the CHMT with support from an action research team (ART). The role of the CHMT is to ensure the application of the A4R conditions during the annual planning and priority setting and in day-to-day decision-making processes. The ART comprises four members of the CHMT and two researchers from research and academic institutions. The two researchers are from the Primary Health Care Institute in Iringa, and the Institute of Development Studies, University of Dar es Salaam. The ART, with support from the rest of the research team, carries out action research. The ART holds meetings every two months to discuss and review the implementation of A4R in the district. Additionally, the researchers hold meetings with the CHMT members every six months to discuss and review the application of A4R conditions. Furthermore, all collaborating research institutions hold annual workshops to review and discuss the experiences of implementing the intervention in the study districts.

Second, throughout the project period, there was close interaction between the ART members and other actors to ensure effective implementation of the A4R approach. The ART members organised sensitisation workshops at the district level to generate enthusiasm, and created expectations not only for the A4R framework but also for the concept of decentralised healthcare planning and priority setting. Stakeholders who were sensitised included: the Regional Health Management Team (RHMT), the Regional Secretariat, the District Health Forum (heads of health facilities), councillors (political leaders), the Chairperson of Health Facility Governing Committees, non-governmental Organisations (NGOs), faith-based organisations (FBOs), community-based organisations (CBOs), and the media.

**Figure 1:**
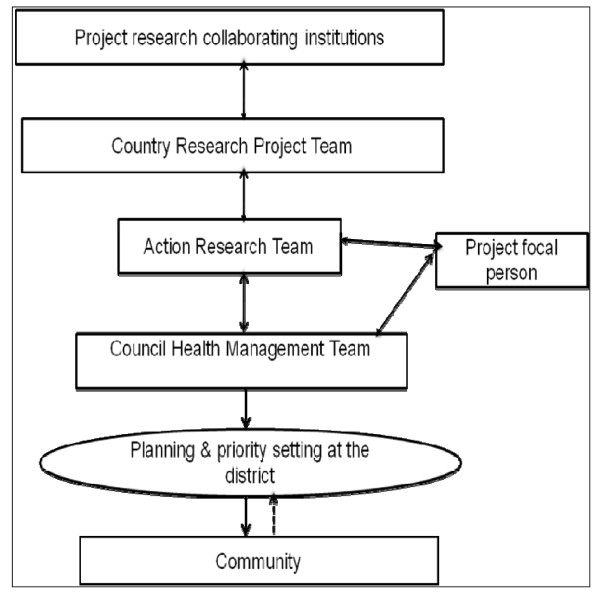
**Relationships of key actors in the implementation of A4R in Tanzania**.

Third, at the request of CHMT members to have a person stationed at the district, in November 2008 the REACT project recruited a focal person who was positioned full-time in the district to observe and facilitate the implementation of A4R. The role of the project focal person included: documenting events related to the implementation of A4R in the district, attending the CHMT management meetings to observe the actual application of A4R in day-to-day decision-making processes, coaching CHMT members on A4R concepts and their application, and capturing the reactions of different stakeholders to the implementation of the A4R framework in the district. Figure 1 illustrates structures and relationships of the key actors in the implementation of A4R in Mbarali district.

### Study design

With a view to capture the complex process of change, realist principles were adopted, which are concerned with illuminating not only the context in which the intervention is implemented, but also the mechanisms of that intervention, as well as its outcomes [24]. The main analytic challenge in this study was not to determine whether or not the A4R framework 'worked', but to find out how the implementation of the A4R was shaped, enabled, and constrained by the interaction between the context (the study's organisational setting and external constraints, including prevailing policies and guidelines) and mechanisms (the stakeholders' ideas about how the change will be achieved through an intervention) [25].

**Figure 2:**
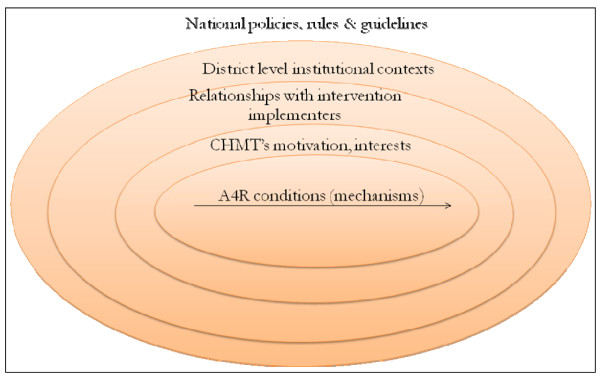
**Interaction between mechanisms of the intervention and different layers of contexts (Modified from Pawson 2006: 32)**.

Ray Pawson [26] has identified four layers of contextual factors that shape the implementation of the social programmes: the individual capabilities of the key actors; the interpersonal relationships supporting the intervention, including lines of communications in the organisation; the institutional settings (culture, informal rules, routines); and the wider contexts (national policies, rules, guidelines) (Figure 2). In line with this understanding, this paper seeks to depict how various layers of contexts have facilitated or constrained the implementation of the A4R intervention in Mbarali district. Conducting the study at this relatively early stage of the project implementation may provide an indication of how the innovation will be integrated into the district health systems and possibly pinpoint the stakeholders' concerns, thereby illuminating areas that will require special attention in fostering sustainability of the innovation that is A4R.

While adopting the realist principles (context-mechanism-outcome), the analysis in this study was primarily focused on the complex interaction between mechanism and contexts. Context matters because the adoption and integration of a health intervention into a health system, and its sustainability, largely depends on a number of contextual factors. Given the fact that this study was conducted two years after the active intervention period in the district, we thought it was premature to assess priority-setting outcomes at this stage. Nevertheless, efforts were made to document process changes and are presented in the results section.

### Data collection techniques

This study adopted a wide range of methods to explore the factors that have influenced the implementation of the A4R intervention in Mbarali district. These included: non-participant observation in the planning meetings, scrutiny of policy, guidelines and project implementation documents, and individual interviews with key stakeholders.

First, from November 2008, the project focal person participated in the priority-setting exercise. Participant observation notes were taken during all priority-setting meetings and sensitisation workshops. The focal person also documented events related to the implementation of A4R in the district and produced monthly reports. The monthly reports also captured the reactions of different stakeholders to the implementation of the A4R framework in the district. Other documents analysed included minutes of the ART and ART/CHMT meetings, and reports from the sensitisation meetings.

Second, the organisational setting and contextual factors surrounding the implementation of the A4R framework were also examined through a review of relevant written documents such as planning guidelines and internal criteria on which priority-setting decisions were based. These documents provided a perspective on the overarching regulations and guidelines from the national government that affect decision making at the district level.

Third, individual interviews were carried out with different categories of actors and stakeholders in the district between January and February 2010. An interview guide was developed and consisted of a series of questions asking respondents to describe factors that facilitated or impeded the adoption and implementation of A4R in the district. Respondents were also asked to identify changes in the planning and priority-setting process over the previous two years. Consistent with qualitative research methods, an open stance was maintained, probing into emerging themes and seeking clarification when necessary. In order to cover a wide range of views of different actors in the district, a purposive sampling technique was used. In total 20 interviews were carried out (Table 2).

**Table 2:** Data sources

	Source of data	Quantity of data
1	Documents	Nine minutes of the ART
		Three minutes of the ART/CHMT
		Three sensitisation reports
		Planning guidelines
		Health policy and strategic plans

2	Field notes	Three observation reports from the planning meetings
		Ten monthly observation reports

3	20 Individual Interviews	Seven members of CHMT
		Two local government officials
		Three members of user committees and boards
		One member of an NGO (advocacy group)
		Two heads of a health facility (health centres)
		Five health workers at the district hospital

### Data analysis

This study adopted the thematic framework approach in which data were classified and organised according to key themes, concepts, and emergent patterns [27]. Interview transcripts and participant observation notes were entered into QSR Nvivo 8 software for storage, coding, text search, and retrieval. The first author developed the code manual based on the research questions, and on the three core components of the realist principles: context, mechanism, and outcome. Next, the first author, in collaboration with two co-authors, read through the transcripts of each interview and identified responses relevant to the main questions raised by the study. Using Nvivo 8 software, data were coded to initial themes. Similar to other qualitative analysis methods, subsequent rounds of analysis led to a refined set of themes and patterns. Thereafter, data were sorted and grouped together under patterns that were more precise, complete, and generalisable [28]. As patterns of meaning emerged, the authors searched for similarities and differences. Finally, data were summarised and synthesised, retaining (as much as possible) key terms, phrases and expressions of the respondents. After this analysis, data were triangulated to allow comparison across sources and different categories of stakeholders. The careful and systematic process of analysis and reflection served to ensure rigour in the analysis [29].

### Ethical issues

The research was approved by the University of Dar es Salaam. The research clearance was presented to the regional and district authorities who approved the study in their areas. Oral informed consent was obtained from all study participants and they were free to withdraw from the study at any time they wished. All the interviews were recorded with the permission of participants and the resulting recordings and transcripts were stored confidentially.

## Results

The next section presents the principal empirical findings organised broadly around the mechanisms of change, which were driving the efforts to improve priority setting in the district. These mechanisms were made explicit in the project's implementation documents. Using these mechanisms, the key enabling and constraining factors are discussed.

### Mechanism one: Using relevant reasons/principles in the priority-setting process

A core principle of the A4R framework is that priority-setting decisions should be based on evidence, reasons, and principles accepted by the stakeholders as relevant for meeting health needs fairly in their context. It was assumed that the use of relevant, explicit principles would improve the quality of decisions and thereby enhance public confidence.

There were efforts by the CHMT to use national guidelines. Planning guidelines require that district health priorities be identified based on locally available epidemiologic data and health service statistics. The sources of evidence that were used included the district Health Management Information System (HMIS), which is based on data collected at health facilities (hospitals, health centres, dispensaries, and village health posts). This data includes cases of notified diseases, deaths, and births, as well as data on the activities of the community health workers.

However, a majority of CHMT members reported that their efforts to use guidelines and evidence were hampered by interference from higher authorities and insufficient information. CHMT members argued that the priorities of the higher authorities (central government) influence the priorities that the CHMT gives to particular areas of health policy. One CHMT member claimed:

'...the guidelines stop us from doing most of the things we would like to do. For example, the government usually requires us to do the things it considers important, even when we've our own plans. At times the things prescribed by the government are not of any importance to the district. Even so, we include them in our plans because the government has decided that they should be carried out.'

A vast majority of the CHMT members also reported lack of reliable monthly and quarterly reports of data from the health facilities. There was a weak link between the CHMT and other committees, such as health centre, dispensary, and ward health committees, which were responsible for supervising the collection of information at their respective health facility.

The CHMT members viewed the identification (collection) of cases of notified diseases, deaths, and births, as well as data on the activities of the community health workers from the catchment areas as a valid, robust, and relevant way of bringing a wide range of relevant reasons into the planning and priority-setting process. It was evident that the CHMT had, since the 2009 planning year, undertaken a number of initiatives to promote the involvement of stakeholders in the process of identifying priorities. First, CHMT members took the initiative to write letters to the catchment areas (district hospitals, health centres, and dispensaries) so that they could identify their priorities and submit them to the District Medical Officer. Second, CHMT members, in collaboration with the REACT project focal person, visited twelve villages to solicit priorities from the community. Attempts were also made by the CHMT to consult hospital staff in an effort to identify hospital priorities, which was summed up as follows by one respondent:

'Since last year we have been involved in identifying priorities. We are asked to identify our priorities at the departmental level. We then send the priorities identified to the CHMT.' (Interview with a health worker)

There was general feeling among the CHMT members that increased involvement of stakeholders in the planning and budgeting process had resulted in more responsive management:

'I would say there are changes. The first change is the planning team itself to be very alert on the priorities set and on people who are unfair in identifying priorities.... So the aggressiveness of the planning team is in itself a noticeable change. In the past, the situation was that the chairperson proposes and the rest remain quiet.' (Interview with a CHMT member)

'The REACT project has opened our eyes. We have now gained confidence and we are able to argue firmly in front of the chairperson.' (Interview with a CHMT member)

It was observed in the 2009/2010 planning and budgeting process that members were given chance to raise issues and engage in discussion, though the chairperson appeared to dominate the discussion and had influence on the final decisions.

There was clear evidence to show that involvement of health workers has increased their awareness and understanding of the planning and priority-setting process. One respondent commented:

'There are significant changes. In the past, only a few people used to determine those priorities and we knew nothing about the process of identifying priorities. But since 2009, there has been greater involvement of the people. Now the process begins at the departmental level and then we move to the CHMT level.' (Interview with a health worker)

By contrast, members of the user committees and boards were not really involved in the planning and budgeting process. They had also not yet been reached as comprehensively as intended through the A4R approach. This is partly because the chosen initial focus for the application of A4R has been predominantly within the CHMT and at the district hospitals aiming, with time, to increasingly include health facilities and communities. This group was generally not satisfied with the district planning and priority-setting process; it felt that while the planning and budgeting process was meant to be participatory, in practice this was not the case. While members of user committees and boards expressed an interest in being more involved in the planning and priority-setting process, some CHMT members felt that the public did not have the knowledge, skills, and experience to effectively contribute to priority-setting decisions.

The CHMT's motivation to engage multiple stakeholders in planning and priority-setting process was partly influenced by the frequency of meetings with researchers, as well as by the existence of the project focal person in the district:

'The REACT people educated us on the importance of community participation in identifying priorities because health priorities are not simply medicine and facilities. Then we decided that the committees should sit together with villagers and identify their priorities.' (Interview with a CHMT member)

'In cooperation with the REACT district focal person we decided to plan village visits in order to meet with the community and know their priorities. We were very successful because the villagers told us so many things some of which we later on incorporated into the revised version of the district health plan of 2009/2010.' (Interview with a CHMT member)

However, all CHMT members reported that participatory structures that could be used to steer stakeholder engagement were not functioning well due to lack of incentives, limited resources, and a low level of awareness of their roles and responsibilities. Interviews with user committees and boards confirmed that many board members did not know what was expected from them.

**Figure 3:**
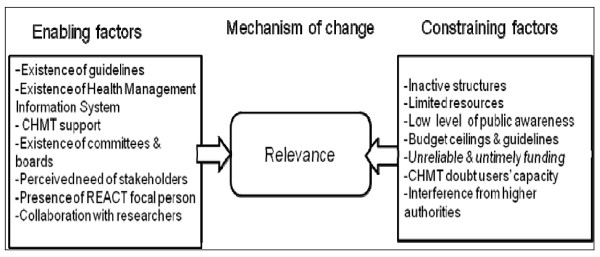
**Realist analysis of attempts to use relevance principles in priority setting**.

The vast majority of CHMT members also reported that their efforts to engage multiple stakeholders were constrained by the delay in the disbursement of funds by the central government. Additionally, planning guidelines and budget ceilings imposed by the national government, as well as interference from higher authorities, were frequently mentioned by almost all CHMT members as obstacles to stakeholder involvement and use of guidelines. Figure 3 summarises factors that both facilitated and constrained the use of relevant principles in the priority-setting process.

### Mechanism two: Publicising priorities to the stakeholders

The second important mechanism of change is publicity. Publicity requires that decision makers in priority-setting contexts should publicise priorities and the reasons for their decisions so that stakeholders, including the public, can understand the value choices involved and can assess whether the relevant procedures are being followed. The A4R intervention assumes that publicity would offer staff and members of the community better access to information on decisions pertaining to them. Better access to information increases 'voice' and allows stakeholders to exert more effective pressure on decision makers, resulting in responsive and fair management.

There were efforts made to disseminate priorities to the health workers, as well as to the public. There was general feeling that district health priorities had become readily accessible to the members of the CHMT and hospital workers. The district priorities were communicated to program leaders and other hospital staff through staff meetings. Priorities were also translated into Kiswahili (the native language) and were pinned on the notice board at the district hospital, district council offices, village council offices, ward executive offices, health centres, and dispensaries (Table 3):

**Table 3:** A sample of district health priorities published on the notice boards

Intervention	Activity	Sources of funds	
		**Block grants**	**Basket funds**
	
	To conduct 36 monthly outreach clinics by 36 health workers	150,000	4,320,000
	
Reproductive and Child health	To conduct nine monthly mobile clinics by four health workers		5,940,000
	
	To conduct training on IMCI for 20 health workers for 14 days		10,085,400

Non- communicable diseases	To procure drugs/supplies for treatment of diabetes, hypertension, injuries		5,354,000
	
	To procure equipment for non-communicable diseases		7,040,000

	To conduct training for three clinicians on emergency oral health care for ten days		2,204,000
	
	To procure two emergency extraction forceps and two pressure cookers/autoclaves		330,000
	
Other diseases	To conduct distribution of zithromax drugs and other supplies/equipments for trachoma mass treatment once per year	575,000	
	
	To conduct training for two days on zithromax treatment		6,237,000

	To collect two water and food samples twice per year for laboratory analysis in Dar es Salaam		3,320,000
	
Health promotion	To collect and dispose of solid waste from six refuse bays	3,480,000	
	
	To conduct a village health competition on environmental health sanitation (5/6/2009) in 80 villages		4,260,000

	To conduct training in 30 health facilities about ILS & forecasting and quantification of medicine for three days	4,054,200	
	
Organisation	To pay extra duty allowance to 20 staff monthly	10,800,000	
	
	To conduct a district health forum for health staff, two times per year for five days		12,271,000

'I would say there are significant changes. Starting from 2009 we have seen hospital priorities displayed on notice boards and in offices. In the past, even the content of the district health plan was not usually known. You would just be told that there was going to be a seminar or training but you would never know what the plans were and whether they were implemented or not.' (Interview with health worker)

'The CHMT has realised the necessity of making the priorities known to patients. The patients read them on the notice boards. Sometimes they ask us for clarification. Indeed, many people are happy about this system and want it to continue.' (Interview with health worker)

However, a majority of the members of the user committees and boards that were interviewed had very little understanding of what the process entailed, and they were not satisfied with the district priorities. Respondents felt that there was a lack of commitment on the part of district health authorities to ensure community participation in the planning and priority-setting process. Further, a low level of awareness and understanding of the district health planning process impinged on their ability to question decisions, summed up thus by one respondent:

'I know that it is my right to see district health plans, since I am among the stakeholders in the health sector. But since we have never been involved we can't enquire. You know something which you don't know is like a totally dark night.... At present, despite being the chairperson of the committee, I don't know the priorities of our hospital.' (Interview with a member of user committee)

**Figure 4:**
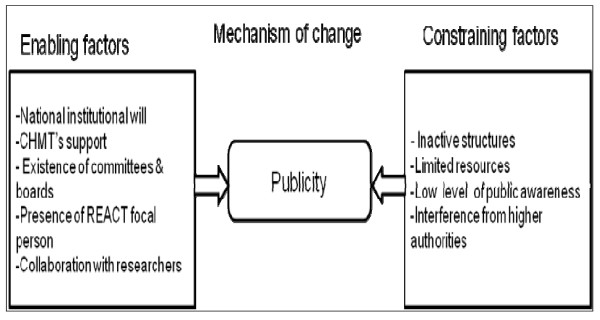
**Realist analysis of attempts to implement publicity**.

Figure 4 shows the contextual factors that influenced efforts to use explicit process and disseminate priorities to the stakeholders.

### Mechanism three: Opportunity to revise and improve decisions over time

The appeals/revision mechanism is intended to have three roles: the appeals/revision mechanism should give members of the planning team and the public a form of a fair process, through which to reverse adverse priority-setting decisions; appeals should give participants an opportunity to air their point of view in the planning and priority-setting process; and appeals should show respect for those who disagree with a particular decision, and provide them with a way of engaging with decision makers. The intended ultimate result is improved quality of decision making, as well as more attention to ensuring the correct implementation of decisions.

At the time this study was being carried out, a formal appeals mechanism had not yet been institutionalised. Procedurally, in the implementation of the REACT project in the district, ART members had started with the relevance and publicity conditions of A4R. This was a step in the process to facilitate the full introduction of appeals and enforcement conditions with other actors beyond the health teams and in communities. The CHMT, in collaboration with the ART, had begun developing an appeals mechanism at the district hospital, through which hospital staff would be able to voice their opinions, views, and concerns on publicised health priorities and management activities.

There was a general feeling among CHMT members that their involvement in planning and priority setting had increased over the past two years. The CHMT members reported that they were now able to appeal against solitary DMO decisions:

'As days pass by there are gradual changes. In the past very few people dominated the meetings. But currently there is room for other members to air their opinions.' (Interview with a member of CHMT)

This was a first step in creating an acceptance of the principle of appeals, and necessary as part of the learning process of becoming responsive to appeals from other actors and the communities.

In addition, the CHMT had started initiatives to publicise priorities on the notice boards at the district hospital, with the intention of getting feedback from relevant stakeholders. CHMT members also had requested that health workers at the district hospital comment on the hospital priorities that were pinned on the notice board. Further, CHMT members, in collaboration with the REACT project focal person, had disseminated priorities to twelve villages in the district, and had requested villagers to give their points of view.

Dissemination of priorities had no obvious effect on appeals/revisability. Based on the interviews, there was low response from the stakeholders regarding the priorities that the CHMT had identified and included in the district health plan:

'Beginning from last year (2009) after the completion of the district health plan we display a summary of the priorities on notice boards at the hospital, in health centres and in ward and village offices. We went even a step further by writing letters requesting health workers and the public to bring their comments. However, we are unhappy because we have not received any feedback as of now.' (Interview with a CHMT member)

'Last year (2009) we started to send summaries of the priorities to 12 villages and they were displayed on the notice boards. The problem that ensued was feedback from the public and other stakeholders. The public and staff did not provide any feedback even after reading on the notice boards.' (Interview with CHMT member)

When the CHMT was further probed as to why stakeholders did not comment on the priorities that were publicised, the common response was that this was a new culture and a majority of the public was not aware of their rights:

'This is a new phenomenon which we started in 2009, the citizenry have not been sensitised to know that this is their right and it is a normal thing. The community needs to know that they have a chance to give their opinions in order to improve the priority-setting process.' (Interview with a CHMT member)

In contrast to this, however, observations revealed that there was fear among the stakeholders to comment on the district health priorities, and the interviews with hospital workers seem to support this view. The district hospital workers stressed that they were invariably hesitant to make any comments in writing, because their handwriting could be identified by the district hospital leadership.

**Figure 5:**
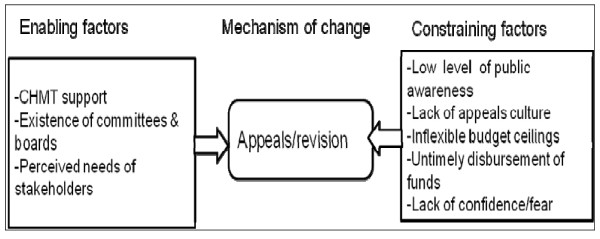
**Realist analysis of attempts to implement the appeals/revision mechanism**.

Further, a majority of those interviewed felt that an appeals mechanism was not feasible in their context. Respondents mentioned low levels of public awareness, a lack of an appeals culture, and inadequate public participation in the priority-setting process as the main barriers to the appeals mechanism. The fact that funds are unpredictable and often earmarked only for certain purposes was also seen as an obstacle to the appeals mechanism. CHMT members argued that resource allocation ceilings do not provide room for reallocation after the district health plans have been approved by the MoHSW (Figure 5). If funds are also reduced or delayed, the district plans no more match main needs, and priorities are revised implicitly or *ad hoc *without time or adherence to any inclusive process.

### Mechanism four: Strengthening enforcement in priority setting

The A4R approach to priority setting requires that there must be public or voluntary regulation of the decision-making process to ensure that relevance, publicity, and appeals mechanisms are met. A review of documents indicated that the central government had put in place rules, regulations, and laws to facilitate and oversee planning and priority setting at the district level. According to the decentralised healthcare framework in Tanzania, the Full Council, the CHSB, and Health Facility Governing Committees (HFGCs) are supposed to oversee and scrutinise district health plans and budgets to ensure that they meet and address local health priorities.

To get the project underway, ART members organised sensitisation workshops at the district level to generate enthusiasm and create expectations not only for the A4R framework but also for the concept of decentralised healthcare planning and priority setting. The A4R members also held sensitisation workshops and participatory discussions with user committees and boards towards understanding A4R conditions and their application in the district-level planning and priority-setting process.

**Figure 6:**
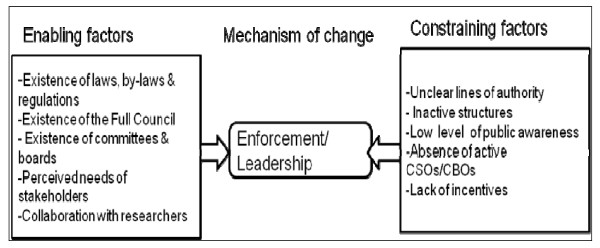
**Realist analysis of enforcement mechanisms in the district**.

However, a majority of respondents reported that efforts to strengthen enforcement mechanisms were hampered by a number of contextual factors (Figure 6). First, the CHSB, despite having authority, did not have the capacity to scrutinise and oversee plans and budgets prepared by the CHMT. Second, members of the board met too infrequently, had insufficient resources, and were not able to fulfil their responsibilities, as expressed by the following respondents:

'....meetings are not held as they should. Our leaders say they can't convene meetings frequently due to financial constraints. If we were holding meetings quarterly, for instance, we would perhaps have a chance to discuss the priorities.... As a result we usually receive reports on things that have already been implemented.' (Interview with a member of CHSB)

Third, oversight institutions had no way to get information about community priorities and so could not assess whether district health plans really reflected and addressed district health priorities. Fourth, members of user boards and committees had insufficient knowledge and experience to oversee priority-setting activities in the district. For instance, the relations between the CHSB, CHMT, and HFGCs were not always as stipulated in law. In fact, the CHSB is mostly bypassed or ignored by the CHMT:

'As far as I know, the district health plans were supposed to be brought to the CHSB for approval before being taken to the Full Council. But the truth is that plans have never been brought to CHSB for approval.' (Interview with a board member)

Further, it was evident that accountability to communities or lower levels of government did not exist. The district had inadequately developed grassroots umbrella organisations that could play the particularly important role of holding district health managers accountable for their plans and decisions.

## Discussion

This study aimed to explore how the A4R innovation has been shaped by individual, organisational, and wider institutional contexts. In the context of low income countries, a few empirical studies have used A4R as a conceptual framework to evaluate priority-setting and decision-making processes [22,30-32]. In addition, another study has recently compared the elements of fairness described in the A4R framework to the elements of fairness as perceived by decision makers [33]. However, no study has, until now, monitored and evaluated the implementation of the A4R framework in low income countries. This study has revealed a number of individual and institutional factors that have influenced the implementation and integration of the A4R intervention into district health systems.

### Individual capacities and interpersonal relationships

First, collaboration and interaction between researchers and practitioners were found to be critically important. The Primary Health Care Institute (PHCI) had in the role of supporters to district health capacity building established a long working relationship with the study district, which facilitated the adoption and implementation of A4R. Additionally, frequent meetings between the researchers and district health decision makers seemed to have increased the level of trust and receptivity to the adoption and implementation of the A4R innovation.

These findings reiterate the importance of supporting collaboration between researchers and implementers in practical settings. Developing social networks between the decision makers and researchers to build trustful working relationships is imperative for fostering the adoption and implementation of innovations in healthcare settings. In addition to formal collaborations, informal networks in the form of friendly relationships among researchers and decision makers are also imperative in linking research and policy, and effecting policy change. Further, a more permanent involvement of researchers in policy and planning efforts, can promote better use of research-based knowledge, but for this to happen there probably needs to be a clearer definition and acceptance of such roles from both research institutions and implementing organisations.

Second, the importance of having a project focal person dedicated to the implementation of the intervention became evident in this study. In Mbarali district, the project focal person who was stationed full-time in the district became part of the implementation team, and was perceived as an expert in the A4R approach to priority setting. A recent study, carried out in other settings, highlighted the significant role of project technical persons in scaling-up and integrating interventions into district health systems [34]. However, to facilitate ownership and integration into local systems, the external project lead person should only be involved in the early stages of the implementation. More recently, a study from Tanzania indicated that, while an external technical assistant was explicitly intended to build capacity for integration into district activities, their existence have actually been counterproductive as district officials felt that they lacked ownership in the implementation process [34].

Furthermore, in this study, the receptivity and attitudes of CHMT members, local government officials, as well as the user committees and boards, towards the A4R intervention proved to be one of the most important factors in fostering its implementation. The CHMT had invested a considerable amount of money and effort to identify the relevant internal and external stakeholders, and to involve them in the planning and priority-setting process. The CHMT's intention to implement the A4R intervention was partly hampered by the infrequency of meetings with researchers.

### District-level organisational settings

While individual and interpersonal factors were important, favourable conditions in the organisational settings also contributed significantly to the implementation of the A4R intervention. The presence of participatory structures under the decentralisation framework was the main organisational factor that facilitated efforts to promote stakeholder participation and transparency in the district.

In contrast, however, infrequent meetings at the grassroots level (such as village council meetings, village general assemblies, and health facility governing committees), limited financial resources, and a low level of awareness among health workers and the public, all served to impede the implementation of the innovation. A fear and a lack of confidence among health workers and the public made it difficult for them to comment on district health priorities.

There is an urgent need for health workers, as well as dispensary and health centre committees and boards, to thoroughly understand the process, and thereby influence decision makers. One of the tools for empowering the user committees and boards is provision of information, more so if they are involved in its collection. Maximising the benefit of channels of influence, may require strategies such as: popular education; building argumentation, advocacy and lobbying skills; and informing staff in the health facilities and village health governing committees about their rights and policies about which they are being consulted [35].

### Wider (national) settings

Whilst local contextual factors were important, national health policy guidelines and procedures also influenced the implementation of the A4R intervention in the district. The central government's call for partnership in district-level planning and priority setting, and the existence of planning guidelines, were the main factors that facilitated the adoption and implementation of the A4R intervention in the district.

In contrast, existing guidelines and procedures in the district were viewed as a barrier to a more inclusive and accountable type of priority setting. The CHMT argued that planning guidelines and budget ceilings imposed by the national government provided very limited room for the district to plan its activities. The CHMT believed that a lot of money was allotted to priorities that were not very critical in the district, while priorities that were of great importance to the district got insufficient funding. The findings of this study suggest that there are still inconsistencies within the district health management systems with respect to resource allocation and funding processes. This means that although a district may need to reallocate more resources, such a move may be constrained when it comes to implementation of interventions, due to restrictions and conditions imposed by resource allocation processes within the basket system. However, national guidelines could be an important tool for effective decentralisation. Given the weak accountability mechanisms at the district and grassroots levels, guidance is needed on the criteria to be debated in the priority-setting and resource allocation processes. Decentralisation may also become problematic if local decision making on how to use resources is left without guidance on citizen rights and local-level responsibilities. Even if financing were to be distributed equitably, local decision makers may choose to use resources in a way which could increase or decrease inequity in access to care. Similarly, local priorities may be at odds with national policy priorities. Nevertheless, it is important that such guidance does not impose new outside criteria that are not at least locally adapted. A4R is intended to facilitate a process that can support fair and sustainable solutions to such dilemmas between actors at district levels, as well as with higher-level authorities. This wider potential remains to be seen.

### Strengths and limitations of the study

Analytically, this study adopted a realist evaluation approach because healthcare organisations are complex. Given the focus of the realist evaluation in uncovering what works, for whom, and under what circumstances, its application to this research was valuable. The findings presented in this study offer insights on how the A4R intervention was shaped, facilitated, and constrained by contextual factors. Such analysis helps to overcome the limitations of traditional case studies in explaining change of the intervention in an open-system setting [26].

The study was limited by its participants. While an effort was made to sample respondents from different levels of decision making in the district, the views and results from the study are not generalisable to other stakeholders. The study setting was only one district and represented the perspectives of a relatively small number of participants. However, even though generalisability was not the intention, the rich description this study has presented still provides a valuable contribution to the knowledge base of priority setting in resource-poor countries.

## Conclusion

This study aimed to explore how the A4R innovation was shaped by individual, organisational, and wider institutional contexts. This study supports the idea that a greater involvement and accountability among local actors through the A4R process may increase the legitimacy and fairness of priority setting decisions. Support from researchers in providing a broader and more detailed analysis of health system elements, and the socio-cultural context, could lead to better prediction of the effects of the innovation and pinpoint stakeholders' concerns, thereby illuminating areas that require special attention to promote sustainability.

Furthermore, the study suggests a need for building strong and effective organisational leadership as an important factor in the successful implementation and sustainability of the A4R approach to priority setting in low income countries. In building the leadership capacity of district healthcare leaders, there is a need to go beyond the skills of medical practitioners to promote the skills of planning, negotiation, lobbying, data management, governance, and accountability to make district health systems effective.

## Competing interests

The authors declare that they have no competing interests.

## Authors' contributions

All five authors contributed to the original design of the study. SM carried out the data collection. SM, AKH and JB analysed the data. SM drafted the manuscript and all authors contributed to the revising of this manuscript. All authors read and approved the final manuscript.
